# From molecule to oblivion: dedicated brain circuitry underlies anesthetic loss of consciousness permitting pain-free surgery

**DOI:** 10.3389/fnmol.2023.1197304

**Published:** 2023-05-25

**Authors:** Mark Baron, Marshall Devor

**Affiliations:** ^1^Department of Cell and Developmental Biology, Institute of Life Sciences, The Hebrew University of Jerusalem, Jerusalem, Israel; ^2^Center for Research on Pain, The Hebrew University of Jerusalem, Jerusalem, Israel

**Keywords:** anesthesia, consciousness, loss-of-consciousness, mesopontine tegmentum, MPTA

## Abstract

The canonical view of how general anesthetics induce loss-of-consciousness (LOC) permitting pain-free surgery posits that anesthetic molecules, distributed throughout the CNS, suppress neural activity globally to levels at which the cerebral cortex can no longer sustain conscious experience. We support an alternative view that LOC, in the context of GABAergic anesthesia at least, results from anesthetic exposure of a small number of neurons in a focal brainstem nucleus, the mesopontine tegmental anesthesia area (MPTA). The various sub-components of anesthesia, in turn, are effected in distant locations, driven by dedicated axonal pathways. This proposal is based on the observations that microinjection of infinitesimal amounts of GABAergic agents into the MPTA, and only there, rapidly induces LOC, and that lesioning the MPTA renders animals relatively insensitive to these agents delivered systemically. Recently, using chemogenetics, we identified a subpopulation of MPTA “effector-neurons” which, when excited (not inhibited), induce anesthesia. These neurons contribute to well-defined ascending and descending axonal pathways each of which accesses a target region associated with a key anesthetic endpoint: atonia, anti-nociception, amnesia and LOC (by electroencephalographic criteria). Interestingly, the effector-neurons do not themselves express GABA_*A*_-receptors. Rather, the target receptors reside on a separate sub-population of presumed inhibitory interneurons. These are thought to excite the effectors by disinhibition, thus triggering anesthetic LOC.

## Introduction

Only recently has consideration of consciousness begun to lose its stigma as an appropriate topic for psychologists and philosophers, but not for biologists. When all is said and done, barring theories contingent on the supernatural, conscious experience is a biological phenomenon that undoubtedly evolved following Darwinian principles with the emergence of increasingly complex organisms. It is realized by neural processes that play out in the ∼1.3 kg of gray matter between our ears. But how computational processes in the brain generate its “experiential” (subjective) color, and that of its congeners including motivation, emotions, pain and will, are entirely unknown. At present, there is a solid barrier between the unimpeded progress in understanding things like pattern recognition, nociception and the generation of complex movement patterns, including their implementation in electronic devices, and general puzzlement when it comes to the experiential. Our strategy in approaching this phenomenon is strictly biological. It begins with molecules, general anesthetic molecules, that are capable of radically altering consciousness, eliminating it completely for a time and sending the “me” inside transiently into oblivion. We then ask where such molecules bind and act: the brain locus(i), the cellular locus and the molecular locus [receptor type(s)]. The next aim is to reverse-engineer the associated circuitry, tracing the synaptic outputs and inputs of the neurons that effect changes in brain-state. The hope is that this strategy will nurture the insight, the conceptual leap, that is undoubtedly required to reveal the fundamental mechanism enabling pain and other conscious experiences. Note that we will be referring to the raw “me” that remains unchanged when eyes are closed and ears covered (phenomenal consciousness), not my attending to, or being conscious of, something in particular (Block, 1995).

## Background

### Anesthetic molecules

The first step was made in 1849 with the clinical demonstration by William T. G. Morton that a small molecule, ether, delivered systemically, results in transient LOC, permitting pain-free surgery (we are aware of other contenders for priority in the discovery of general anesthesia). From that moment on surgeons were no longer constrained by the unimaginable suffering of the patient and could perform ever more intricate lifesaving surgeries (Robinson and William, 1946; Fenster, 2002). General anesthesia is defined as reversible coma, a drug-induced state characterized (with some over simplification) by atonia, analgesia, amnesia, and LOC (Brown et al., 2010). For its first 100 years the locus of action was presumed to be the entire brain with the molecular target being membrane lipids, this based on the Overton-Meyer correlation (Overton, 1901; Goodman and Gilman(eds)., 1990). Subsequently, the already well-established concept that GABA receptors (GABA-Rs) are the actual target, for barbiturate anesthetics at least, was sealed in 1978 by a groundbreaking publication by Franks and Lieb (1978). They convinced the community that the molecular targets of general anesthetics, in general, are proteinaceous, probably membrane receptors. The accepted locus of action within the brain, however, the answer to the question: “Where do general anesthetics act?” remained “widespread.” We refer to this as the “wet-blanket” hypothesis of anesthesia.

A caveat, emerging from the work of Joseph Antognini, inserted an interesting modification into the wet blanket hypothesis; that different anesthetic endpoints might be realized at different brain loci. Specifically, based on experiments carried out using goats, Antognini and co-workers used clamps and pumps to separate the vascular blood supply to the brain from that supplying the spinal cord. These experiments suggested that amnesia and LOC are induced when anesthetic drugs are delivered selectively to the cerebral cortex while atonia and analgesia, without LOC, are realized when they are delivered selectively to the spinal cord (Antognini and Carstens, 1998). This “patch-wise” version of the wet-blanket hypothesis retains the key feature of the original. Specifically, anesthetic drugs travel in the circulation to various widely spread cerebral loci at which the individual component parts of anesthesia are executed. There, they act on transmembrane receptors to induce and maintain oblivion. This “patchwise wet blanket theory” remains the most widely held notion of how anesthesia works, the “canonical” concept, in the clinical anesthesia community at least (Rampil et al., 1993; Hentschke et al., 2005; Antognini et al., 2010; Mashour and Hudetz, 2017; Pal et al., 2018; Voss et al., 2019).

### Where do anesthetic molecules act?

A very different concept of brain-state transitioning emerged in the 1940–50’s based on clinical observations on natural sleep, with follow-up using animal models. The key observation was that focal lesions in the posterior hypothalamus and adjacent brainstem can cause prolonged somnolence and coma (von Economo, 1918; Bremer, 1936; Nauta, 1946). The upshot was identification of a zone in the dorsal mesopontine tegmentum that sends signals along ascending axonal pathways to maintain the cortex in an aroused state (wakefulness). This zone and associated pathways constitute Moruzzi and Magoun’s (1949) ascending reticular activating system (aRAS). Although the term “aRAS” is not used much anymore, a fair fraction of subsequent research on sleep has been devoted to defining the specific cell groups and axonal pathways that constitute the aRAS. Today, there is broad consensus that sleep is controlled by circuitry, i.e., dedicated pathways (Lu et al., 2006; Liu and Dan, 2019), although the concept of circulating “somnogens” as a contributor to sleep has not been entirely forgotten (Borbely and Tobler, 1989; Gallopin et al., 2005).

Less well-known is that already in the 1950s Moruzzi and Magoun published evidence that anesthetics acting in the aRAS cause transitioning from wakefulness to LOC (French et al., 1953; Magni et al., 1959), suggesting that dedicated pathways might also contribute to anesthetic LOC. This idea lay dormant for decades, however, until the recent wholesale adoption of sleep circuitry by the anesthesia research community as a basis for the “dedicated pathways hypothesis of general anesthesia.” The idea is that anesthetic agents act by substituting for an endogenous neurotransmitter(s); that they cause LOC by “hijacking” the sleep circuitry (Solt and Forman, 2007; Franks, 2008; Orser and Saper, 2008; Brown et al., 2010; Scharf and Kelz, 2013; Jiang-Xie et al., 2019; Jones, 2020). Support for this hypothesis comes from the realization that quite small brainstem lesions in humans lead to unconsciousness (coma) while quite large cortical lesions rarely do. And when they do, it is usually a result of elevated intracranial pressure causing brainstem damage (Fischer et al., 2016; Posner et al., 2019; Snider et al., 2020; Baron and Devor, 2022). The key difference between the “dedicated pathways hypothesis” and the “wet-blanket hypothesis” is that the primary site-of-action of anesthetic agents is not where the main anesthetic components are realized (mostly cortex and spinal cord), but rather at brainstem targets that modulate the cortex and spinal cord via dedicated ascending and descending axonal projections. This difference has substantial functional consequences (below).

## The mesopontine tegmental anesthesia area (MPTA)

### Microinjection

Our contribution to this problem area began with a systematic search for **where** anesthetics act, the “where” referring to brain locus rather than the molecular target. The starting-point **molecule** chosen for the (where?) search, pentobarbital (PB), is the prototypical exemplar of a GABAergic anesthetic. The strategy was to microinject PB systematically, through surgically implanted guide cannulae, in a 3D matrix covering the entire rat brain (Devor and Zalkind, 2001). *A priori* it was far from certain that there exists a single brain locus at which PB can evoke anesthesia. There might be no master locus for the anesthetic action of PB, several loci might have to be recruited simultaneously, or perhaps only individual sub-components of anesthesia can be evoked by focal drug administration. Moreover, since we are discussing co-opting complex circuitry, it is likely that different anesthetic agents have different actions. Ketamine or isoflurane, for example, might interact with the circuit at different locations than PB (Anis et al., 1983). To improve the odds, in our initial survey study PB microinjections were made bilaterally. The thought was that the endogenous analog of PB, GABA, is normally inhibitory. To activate brain functions stimulation on one side is usually enough, but to extinguish them both sides are usually required (consider seeing and hearing).

**FIGURE 1:**
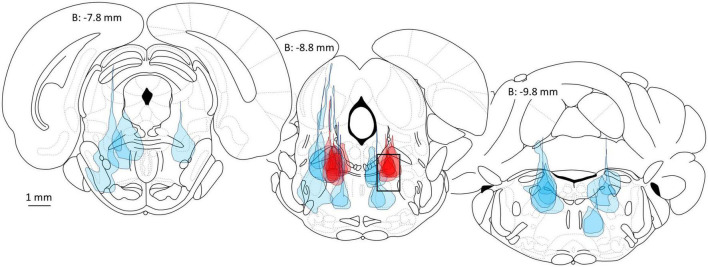
Delivery of the potent GABA_*A*_-R agonist muscimol into the mesopontine tegmental anesthesia area (MPTA) is pro-anesthetic (dark red microinjection patches, 10 left side, 7 right in 9 rats, all are 10 or 20 nL), whereas microinjection elsewhere in the mesopontine tegmentum (light blue patches), or elsewhere in the brain (Devor and Zalkind, 2001) is ineffective. The location of the MPTA is shown by a rectangle in the middle brain section (8.8 mm posterior to bregma, dimensions: M-L 1 mm × D-V 1.5 mm × A-P ∼2.0 mm). Coordinates used for MPTA microinjections are A-P: −8.6, M-L: ±1.3 and D-V: −6.3. All microinjections shown are unilateral, on the side indicated. Figure modified from Minert et al. (2017).

The bottom line of the initial microinjection survey, and several follow-up studies, was the discovery of a single, small bilaterally symmetrical site located in the rat brainstem at which microinjection of minute quantities of a variety of GABA_*A*_-R agonists including PB and propofol rapidly evokes a state of surgical general anesthesia lasting tens of minutes (Figure 1; Minert et al., 2017). Although in the survey saturating concentrations of PB and propofol were used, we later documented that microinjecting clinically relevant concentrations of PB or propofol, the concentrations measured in the CSF during systemic-induced anesthesia, are also pro-anesthetic (Baron et al., in preparation). Microinjections less than 1 mm off-target were ineffective (Figure 1). On-target microinjections evoked a brain state transition that combined atonia, analgesia and a sleep-like encephalographic (EEG) pattern. We have not yet checked for loss of memory formation.

**FIGURE 2:**
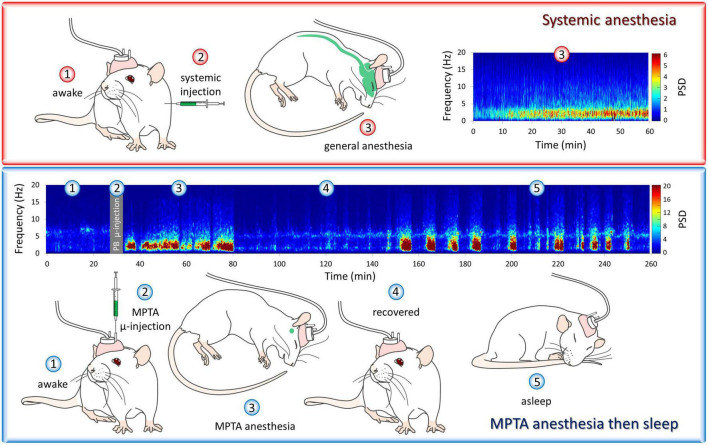
“Paradoxical anesthesia”. The cortical encephalographic (EEG) signature following systemic delivery of pentobarbital (PB) is consistently δ-band dominant **(upper panel)**, while PB microinjected into the mesopontine tegmental anesthesia area (MPTA), unilaterally in this case, yields a very different EEG pattern. This pattern, which is bilaterally symmetrical, resembles the alternating REM-NREM pattern typical of natural sleep **(lower panel)**. We call this “paradoxical anesthesia”. The animals’ behavioral state is illustrated by numbered drawings. These same numbers, shown on the spectrograms, indicate the EEG pattern recorded at the same times. At time-points 1–4 (paradoxical) anesthesia lasting about 50 min is induced by unilateral MPTA microinjection of PB followed by emergence (4, recovery). Subsequently, this rat entered a period of natural sleep (5). Observations from Avigdor et al. (2021). For spectrogram colors the reader is referred to the web version of this article.

An interesting aspect of the EEG signature observed during microinjection-evoked anesthesia is that it features alternating epochs of NREM-like, delta-wave dominant EEG and REM-like (wake-like) EEG. This pattern, which we call “paradoxical anesthesia,” differs markedly from the uniform delta-wave dominant EEG pattern normally seen during systemic-induced GABAergic anesthesia (Figure 2; Supplementary Video 1; Avigdor et al., 2021; Baron et al., 2022). This supports the notion that via axonal projections we are tapping into natural sleep-wake circuitry that is located nearby, within the reticular formation and at a distance. The sustained delta-EEG associated with systemic-induced anesthesia, as well as the thermoregulatory and respiratory suppression typical of clinical anesthesia, appear to reflect off-target drug action. They are not evoked by microinjection even at high drug concentrations, and hence are probably not integral features of anesthetic brain-state switching.

As the effective microinjection locus was reticular and did not correspond to a previously defined nucleus, we named it for its location and putative function, the mesopontine tegmental anesthesia area (MPTA) (Sukhotinsky et al., 2016). The MPTA lies within the much larger aRAS, the zone Moruzzi and Magoun (1949) defined more than 70 years ago. Induction of LOC by exposure of the MPTA to barbiturates has been replicated successfully by others (Voss et al., 2005; Namjoshi et al., 2009). However, Voss et al. (2005) were unable to replicate our observation that LOC can be induced upon microinjection of propofol although they do report anti-nociception as an outcome. We have no obvious explanation of this result, but can add that in the intervening years we have consistently obtained pro-anesthetic effects using propofol and other GABAergic agonists, including at clinically relevant concentrations.

### Model of MPTA function

**FIGURE 3:**
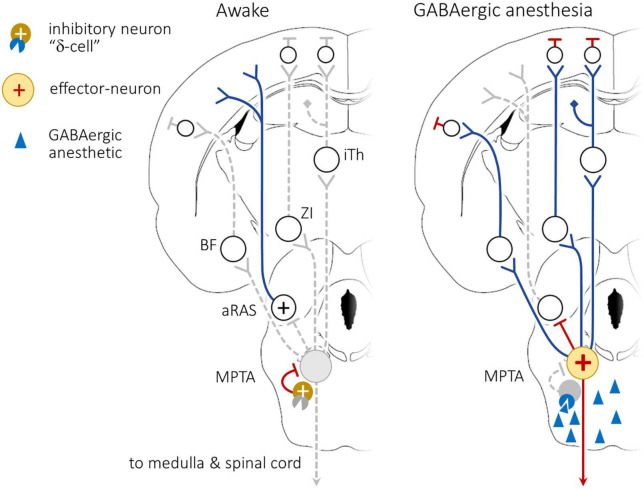
Working model of anesthetic induction following exposure of the mesopontine tegmental anesthesia area (MPTA) to GABAergic anesthetics. **(Left)**: During the awake state tonically active inhibitory interneurons within the MPTA, cells that express GABA_A_δ-Rs (“δ-cells”), strongly inhibit the effector-neurons. Activity originating in aRAS nuclei, perhaps augmented by forebrain arousal nuclei that access the cerebral cortex, maintain wakefulness. **(Right)**: GABAergic agents accessing the MPTA, following delivery by the systemic route, or by direct microinjection (blue triangles) bind to GABA_A_δ-Rs on the δ-cells, inhibiting their ongoing activity. This releases the effector-neurons from tonic inhibition (yellow circle with +), disinhibiting them and allowing them to fire actively. The “brakes” are released. Activity of the effector-neurons now causes widespread functional suppression in the cortex, including altered EEG pattern. This suppression is presumed to be mediated by inhibition of ascending reticular activating system (aRAS) nuclei as well as by recruitment of dedicated ascending pathways that relay through the intralaminar thalamus (iTh), the zona incerta (ZI) and the basal forebrain (BF). There also exists a modest direct projection to the prefrontal (PFC). The model is non-committal concerning the mechanism of cortical inhibition, direct or via intracortical inhibitory interneurons. Simultaneously, activation of descending bulbo-spinal inhibitory pathways, both descending inhibitory pathways to the spinal cord (SC), and pathways relayed through the rostro ventromedial medulla (RVM) induce atonia and analgesia. Modified from Baron et al. (2022). For interpretation of the references to color in the figure, the reader is referred to the web version of this article.

Remarkably, the effects described above are also readily evoked by unilateral MPTA microinjection, hinting that an excitatory rather than an inhibitory process might be involved. Indeed, transient silencing of spike activity in the MPTA by on-target microinjection of the Na^+^ channel blockers lidocaine or tetrodotoxin (TTX), unilaterally or bilaterally, was ineffective. Even permanent cell-selective destruction of the MPTA using ibotenic acid (microinjected unilaterally or bilaterally) did not produce prolonged unconsciousness (coma) although it did have other effects on arousal as described below. These observations formed the basis of a model of MPTA functioning that sees GABA and GABAergics as suppressing the activity of spontaneously active GABA-sensitive inhibitory interneurons. This suppression removes inhibition from putative “effector-neurons” in the MPTA whose ascending and descending axonal projections bring about the individual components of anesthesia (Figure 3; Devor et al., 2016; Baron et al., 2022). The last step in the model has direct observational support. Specifically, Namjoshi et al. (2009) showed that spontaneous activity of spinothalamic tract neurons in the dorsal horn, and their response to peripheral evoked stimulation, is inhibited by focal delivery of PB to the MPTA.

The model explains why inhibition by GABAergic agents differs from inhibition by lidocaine, TTX and lesioning. GABAergics excite effector-neurons (by disinhibition). Lidocaine, TTX and lesioning, in contrast, suppress effector-neurons preventing them from signaling the next node(s) in the circuit. Brain surveys using c-Fos expression as a marker of excitation have revealed that network interactions triggered by normally inhibitory agents like PB yield strong excitation in a number of nuclei in the CNS. Chief among these are the lateral habenular nucleus (LHb), the paraventricular hypothalamic nucleus (PvH), the supraoptic nucleus (SON) and the zona inserta (ZI). Looking at higher resolution large numbers of brain structures, maybe all, include at least some such “anesthesia-on” neurons, neurons that are excited by GABAergic anesthetics (Lu et al., 2008; Abulafia et al., 2009; Yatziv et al., 2020, 2021). It is premature, however, to rule out the possibility that some of this excitation reflects a direct drug action mediated by GABA_*A*_-R expressing neurons in the presence of a reversed Cl^–1^ gradient, rather than by network effects such as disinhibition (Wagner et al., 1997; Ben-Ari, 2002; Price et al., 2005). Although MPTA effector-neurons lack the most common GABA_*A*_-R isoforms, we cannot rule out the possibility of less studied GABA_*A*_-R isoforms being expressed by these neurons (Sinkkonen et al., 2000; Baron et al., 2022).

### Effects of lesioning the MPTA–sufficient and necessary

While block or destruction of the MPTA failed to result in coma as might have been expected, the animals did show distinct phenotypes associated with arousal. First, their response to systemic delivery of the GABAergic anesthetics PB and propofol was markedly reduced. The i.v., dose had to be increased to nearly lethal levels before lesioned animals showed loss of the righting reflex (LORR). The fact that anesthesia can nonetheless be induced at high enough concentrations, despite destruction of the MPTA, indicates that its various components are executed in far-flung areas of the CNS, cortex and spinal cord. This also accounts for the results of Antognini et al. (2010) noted above, that direct exposure of the cortex and spinal cord, separately, at high concentrations, yields component parts of anesthesia (patch-wise wet-blanket concept). In contrast, local exposure of intact MPTA neurons to anesthetics in minute quantities and at relatively low concentrations evokes all of the component parts of anesthesia, via dedicated pathways that act on these far-flung executive regions (Minert and Devor, 2016).

Additional functional changes caused by MPTA lesions are insomnia, a significant increase in awake time at the expense of sleep (REM and non-REM equally), and resistance to LOC induced by hypercapnia, elevated levels of CO_2_ (Meiri et al., 2016; Lanir-Azaria et al., 2018). Together, these observations add to the emerging picture that the MPTA has executive control over brain-state transitions of arousal in general, not just response to exogenously administered pharmacological agents. Interestingly, in MPTA-lesioned animals no change in anesthetic potency was seen using the non-GABAergic anesthetics ketamine, medetomidine and others (Minert and Devor, 2016; Minert et al., 2020). These may act through different mechanisms.

The microinjection survey experiments described above established the MPTA as a singular brain locus for anesthetic induction using GABAergic agents. Unilateral PB microinjections as small as 10 nL, calculated to expose no more than about 1,900 neurons to the drug, proved to be pro-anesthetic. They generate both atonic and analgesic effects mediated by the spinal cord, as well as broad synchronization of the EEG reflecting an action in the cerebral cortex (Namjoshi et al., 2009; Minert et al., 2017; Avigdor et al., 2021). These effects must be mediated by impulse propagation along axonal pathways. Their onset following MPTA microinjection is far too rapid, no more than ∼1–2 min, to be attributed to diffusion to the cortex and spinal cord. Likewise, LOC occurs at doses far too low to be effective after dilution in the systemic circulation. Doses effective when microinjected have no effect when delivered i.v., The wet blanket hypothesis can be ruled out.

The engagement of MPTA neurons thus appears to be **sufficient** to induce anesthetic loss-of-consciousness. We stress, however, that the MPTA is **not** the proverbial “center of consciousness.” Its destruction does not cause unconscious oblivion. It is only a single element in wake-unconscious circuitry, although an important one. We conceive of the MPTA as a “flip-flop” switch that acts on distant structures through a cascade of specific neuronal pathways. One such structure might be the ventrolateral preoptic area (VLPO) a region known, in turn, to modulate the activity of many brainstem arousal nuclei (Lu et al., 2006). Indeed the VLPO has direct projections to the MPTA and nearby nuclei including the pedunculopontine tegmental nucleus (Sherin et al., 1998). The swiftness of brain-state switching mediated by this circuitry is exemplified by the experience of reading a book in bed at night. Who hasn’t abruptly fallen asleep, entering a state of lost consciousness with atonia. The book drops from your hand and hits your knee causing a stimulus that briskly wakes you up, whereupon you seamlessly resume reading the book. Another example is listening to a boring lecture and repeatedly nodding off, like a woodpecker. The “computer” in your head shuts down and re-boots, all within a second or so.

In addition to being **sufficient**, an intact MPTA is also **necessary** for the normal course of LOC induction by GABAergic agents. As noted, bilateral MPTA lesions cause insensitivity to PB and propofol at the usual, clinically relevant doses. We stress, however, that it does not cause the animal to become “immune” to GABAergic agents. Increasing the concentration of drug in the systemic circulation beyond the usual clinical dose eventually leads to LOC and ultimately to death. This is almost certainly due to actions at locations other than the MPTA, for example at medullary respiratory centers, or perhaps in the manner of a wet blanket. To be sure, delivery of very high concentrations of PB or propofol to the MPTA in intact animals does not cause hypothermia, respiratory suppression and death (Devor and Zalkind, 2001; Avigdor et al., 2021).

### Dedicated pathways, collateralization, and dissociations

**FIGURE 4:**
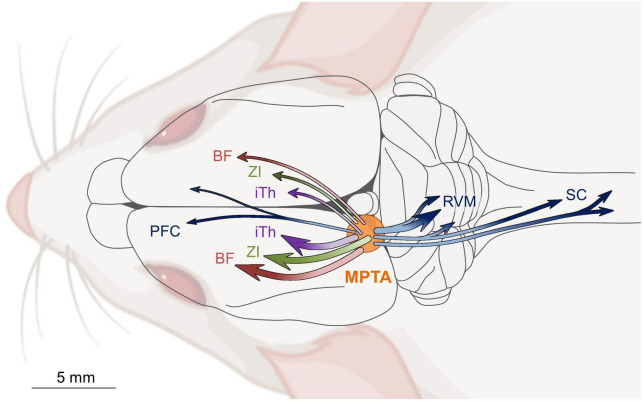
Schematic diagram of ascending and descending projection pathways that originate in the mesopontine tegmental anesthesia area (MPTA) and access the cerebral cortex, and the medulla (RVM) and spinal cord (SC). With few exceptions, independent populations of MPTA effector-neurons project rostrally to terminate, with little collateralization, either ipsilaterally (about 2/3), or contralaterally (about 1/3) in one of three forebrain relay nuclei: intralaminar thalamus (iTh), zona incerta (ZI), or basal forebrain (BF). MPTA neurons with descending projections to the hindbrain and spinal cord show a considerably greater degree of collateralization to multiple targets. Modified from Lellouche et al. (2020).

The axons of MPTA neurons that project to rostral targets are not collateralized. Individual projection neurons send their axons to a single specific distant target, either ipsilaterally or contralaterally, but not both. The most prominent of the ascending projection targets are the intralaminar thalamus, the zona incerta and the basal forebrain. Each of these targets are known to relay their input to wide swaths of the cerebral cortex. There is also a modest direct projection from the MPTA to the prefrontal cortex. Projection neurons that target caudal brain structures show a higher degree of collateralization. The main synaptic targets of these neurons are the rostral ventromedial medulla (RVM) and the spinal cord. A considerable fraction of MPTA projection neurons that innervate the RVM also send collaterals to the spinal cord. Moreover, a majority that projects to one segment of the spinal cord also sends collaterals to other segments (Figure 4; Sukhotinsky et al., 2006; Reiner et al., 2007; Goldenberg et al., 2018; Lellouche et al., 2020; Baron et al., 2022).

We venture that the individual pathways subserve different anesthetic endpoints. Specifically, ascending pathways are likely to mediate amnesia and the widespread synchrony of burst firing that underlies delta-wave EEG and LOC, while the descending pathways subserve analgesia and atonia (Volgushev et al., 2006; Mann and Paulsen, 2007; Franks, 2008; Namjoshi et al., 2009; David et al., 2013; Bharioke et al., 2022). This is consistent with the observation that these four endpoints, although usually engaged and disengaged simultaneously as a syndrome, occasionally dissociate. For example, REM-atonia can occur without LOC in cataplexy or sleep paralysis. Conversely, parasomnias such as “sleep-walking” illustrate the opposite dissociation, LOC without motor suppression. While generating anesthesia including LOC, anesthetic agents do not quench all cortical computations. The classical studies of visual and somatosensory processing in V1 and S1 cortices that uncovered line-detectors, cortical columns etc., were mostly carried out in anesthetized animals (Mountcastle et al., 1957; Hubel and Wiesel, 1959). The selectivity of projection systems associated with brain-state transitions also probably subserve alternating uni-hemispheric sleep, a strategy used by cetaceans (dolphins, whales) and some birds to retain consciousness while at the same time “resting” the cerebrum (Lyamin et al., 2008; Mascetti, 2016). Dissociations have also been observed following MPTA microinjection. For example, 5% lidocaine microinjected into the MPTA suppresses pinch response but not rhythmic pacing, and taurine suppresses nocifensive reflexes and pacing, but not muscle tone (Devor, 2016; Baron et al., in preparation).

### MPTA “effector-neurons”: linking locus-of-action to functional circuitry

The considerations reviewed above go a long way toward answering the question of where in the brain, locus and receptor, anesthetic agents bind and act. Using chemogenetic tools we have recently taken a major step toward linking locus to function. Specifically, we succeeded at introducing into MPTA projection neurons DREADDs, under both human synapsin and CamKII promoters. DREADDs are engineered receptors that allow selective excitation or inhibition of the DREADD-expressing neurons using an exogenous agonist, clozapine-N-oxide (CNO) that is otherwise largely inert (Armbruster et al., 2007). Remarkably, as predicted by our working model (Figure 3), inhibition of these neurons with CNO had no obvious effect, while exciting them was pro-anesthetic (Baron et al., 2022). CNO was transformed into an anesthetic agent. We term the neurons identified in this way “MPTA effector-neurons” as they are the ones that effect brain-state transition when excited.

Since the DREADD construct also causes a red fluorescent reporter marker, mCherry, to be expressed in the effector-neurons, it is straightforward to use immunolabeling to characterize these neurons in terms of neurotransmitter(s) used, receptors expressed and anatomical connectivity. Most effector-neurons are glutamatergic (∼70%) and send ascending and descending projections to the relay nuclei and other targets that we previously identified using anterograde and retrograde tracing (Figure 4). Most, or perhaps all, MPTA projection neurons are effectors and as such have targeting and collateralization properties already established and described above. And as noted, they do not appear to express GABA_*A*_-Rs, at least not the conventional synaptic (γ_2_-subunit expressing) or extrasynaptic (δ-subunit expressing) isoforms. On the other hand, the MPTA contains a large population of small δ-subunit expressing cells. These, rather than the effector-neurons, appear to be the cellular targets of GABAergic anesthetics (Baron et al., 2022).

## Summary and perspective

Surgical anesthesia is a modern invention. The brainstem circuitry upon which anesthetics act must surely have emerged evolutionarily in the context of some natural need(s), presumably including sleep-wake transitions. We believe that GABAergic anesthetics co-opt this endogenous adaptive circuitry by an action on the brainstem MPTA where activity in the effector-neurons leads to LOC and all its components. Like circulating hormones that distribute widely but act on specific target organs, the entire CNS is exposed to circulating anesthetics, but they act to induce LOC primarily in the MPTA. Precisely how the MPTA interacts with established sleep cycle models is still unresolved. MPTA lesions do not prevent rats from falling asleep, but they do alter sleep-wake patterns by shortening total NREM and REM and enhancing wakefulness (Lanir-Azaria et al., 2018). We suspect that other instances of LOC such as fainting, concussion, epilepsy and hibernation also engage the endogenous sleep-wake circuitry (Hayes et al., 1984; Minert and Devor, 2016).

### Dissociating components of anesthesia and sleep

A perplexing fact surrounding LOC under natural circumstances, and anesthesia, is why the high-order functions of memory and conscious awareness, presumably cortical functions, are lost together with the lower functions, presumably spinal, of atonia and analgesia. At some point in the evolution of the sleep-wake circuitry it must have been advantageous and hence adaptive to lose muscle tone and sensory awareness while unconscious. Perhaps the benefit was to avoid enactment of dreams, or to lower one’s head to facilitate or restore circulation to the brain? Or perhaps this pairing served as a defense mechanism against predators (“playing dead,” psychogenic syncope)? Regardless, consciousness and complex motor acts do not necessarily partner. As noted, instances where one occurs exclusive of the other are common, like sleepwalking (somnambulism), where a person can make a sandwich or even drive a car, without any conscious awareness and with NREM sleep-like EEG. Another instance is dreaming, where we have a rich conscious experience often full of action, while fully atonic [R v. Parks, [1992] 2 SCR 871; (Zadra et al., 2013)]. Consciousness and memory formation may also dissociate. Consolidation of fleeting experiences into a long-term form is believed to occur during sleep. Conversely, vividly experienced actions occurring in dreams tend to fade rapidly when we awaken. Irrespective of the evolutionary reasons for the four-way association of LOC, or the occasional dissociation of pain, movement, memory and consciousness, the hardware required is in place. The MPTA features ascending and descending projections where single neurons project to unique functional relays (Figure 4; Reiner et al., 2007; Lellouche et al., 2020).

The presence of a wiring pattern that supports dissociation of the individual components of LOC opens the possibility of exploiting selective dissociations therapeutically. For example, perhaps it might be possible to pharmacologically engage the descending MPTA projections that are responsible for the powerful anti-nociceptive effects of anesthesia without causing sedation or LOC. Correspondingly, it might be possible to awaken certain comatose patients by modulating ascending MPTA pathways.

### Consciousness: neural mechanisms

Advancements in artificial intelligence (AI) have made machine algorithms and even word processors perform so naturally and fluently that humans interacting with them can be forgiven for attributing to them subjective awareness. This is a sort of adult version of kids attributing conscious experience to dolls and puppets. In reality, nothing in the AI software that we know of generates consciousness itself. We infer consciousness based on the expertise of the performance, just as we are amazed by an expert magician who can make an elephant disappear before our eyes. In contrast, insects for example, appear to most of us to be like robots, deprived of any internal subjective conscious experience. Their behavior is “machine-like” and does not resemble what we humans expect from sentient beings. Nonetheless, it is widely presumed that subjective experience did not emerge with humans, but rather appeared earlier in evolution. In what way this phenomenon is adaptive and facilitates survival and propagation of the species more successfully than the philosophers’ unconscious “zombie”, remains elusive (Block et al., 1997; Devor et al., 2015). Whatever the explanation, we have argued based on imaging and other data related to nociception and pain that raw consciousness (phenomenal consciousness), the “me” inside, might have arisen relatively early and be “seated” in the brainstem rather than in the cerebral cortex (Baron and Devor, 2022).

Be that as it may, the observation that anesthesia-induced brain-state transitions are mediated by dedicated brain circuitry with a nodal point in the MPTA constitutes a practical experimental lead into the study of conscious experience itself at the level of molecules, receptors, neuronal types and connectivity. The substrates of subjective experience can be tackled with the tools of biology. Our intuition, however, suggests to us that the experimental path ahead will require more than reverse engineering of the cells and circuits that implement conscious experience in the mammalian brain. There is something missing in our current understanding of the underlying neural computations that will need to be bridged… the explanatory gap (Feinberg and Mallatt, 2019). The history of science, however, suggests that the needed insight, the “eureka moment”, is likely to favor the individual actively grappling with the problem within a conducive experimental framework (Kuhn, 1962). Consciousness is a biological phenomenon. How are ∼1.3 kg of neurons, axons and neurotransmitters able to generate conscious experience?

## Author contributions

MB and MD conceptualized the contribution, revised, and edited the manuscript. MB wrote the first draft. Both authors contributed to the article and approved the submitted version.
